# Real-time investigation of reactive oxygen species and radicals evolved from operating Fe–N–C electrocatalysts during the ORR: potential dependence, impact on degradation, and structural comparisons†

**DOI:** 10.1039/d4sc01553c

**Published:** 2024-05-31

**Authors:** Seth T. Putnam, Joaquín Rodríguez-López

**Affiliations:** a Department of Chemistry, University of Illinois Urbana-Champaign 600 S. Matthews Ave. Urbana IL 61801 USA joaquinr@illinois.edu

## Abstract

Improving the stability of platinum-group-metal-free (PGM-free) catalysts is a critical roadblock to the development of economically feasible energy storage and conversion technologies. Fe–N–C catalysts, the most promising class of PGM-free catalysts, suffer from rapid degradation. The generation of reactive oxygen species (ROS) during the oxygen reduction reaction (ORR) has been proposed as a central cause of this loss of activity. However, there is insufficient understanding of the generation and dynamics of ROS under catalytic conditions due to the difficulty of detecting and quantifying short-lived ROS such as the hydroxyl radical, OH˙. To accomplish this, we use *operando* scanning electrochemical microscopy (SECM) to probe the production of radicals by a commercial pyrolyzed Fe–N–C catalyst in real-time using a redox-active spin trap methodology. SECM showed the monotonic production of OH˙ which followed the ORR activity. Our results were thoroughly backed using electron spin resonance confirmation to show that the hydroxyl radical is the dominant radical species produced. Furthermore, OH˙ and H_2_O_2_ production followed distinct trends. ROS studied as a function of catalyst degradation also showed a decreased production, suggesting its relation to the catalytic activity of the sample. The structural origins of ROS production were also probed using model systems such as iron phthalocyanine (FePc) and Fe_3_O_4_ nanoparticles, both of which showed significant generation of OH˙ during the ORR. These results provide a comprehensive insight into the critical, yet under-studied, aspects of the production and effects of ROS on electrocatalytic systems and open the door for further mechanistic and kinetic investigation using SECM.

## Abstract 2

Hydroxyl radical and hydrogen peroxide are implicated in the poor stability of Fe–N–C catalysts. We use SECM to detect these transient species in real time to evaluate their possible sources and relationship to stability.

## Graphical abstract

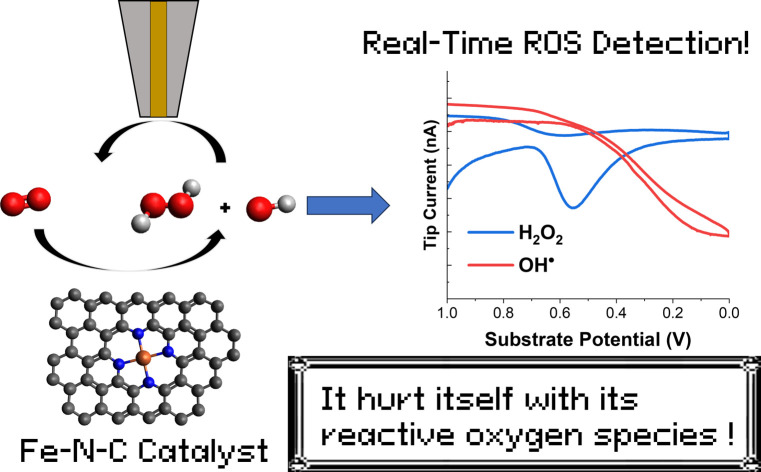

## 1. Introduction

The development of a variety of sustainable energy conversion and energy storage technologies depends on effectively catalysing the oxygen reduction reaction (ORR). Several of these promising technologies include proton exchange membrane fuel cells^1–3^ and metal–air batteries.^4^ Unfortunately, the ORR suffers from slow kinetics, and devices typically need high loadings of platinum group metal (PGM) catalysts for the reaction to proceed at an appreciable rate.^5–7^ However, the high cost of these PGM catalysts is a significant limitation in the adoption of fuel cell technologies.^8^

One promising avenue to reducing the cost and improving the performance of proton exchange membrane fuel cells (PEMFCs) is the development of metal–nitrogen–carbon (M–N–C) catalysts.^9–11^ These catalysts were first introduced in 1964 when Jasinski found metal phthalocyanines to be active towards the reduction of oxygen.^12^ The use of inexpensive, earth-abundant elements (*e.g.*, iron, nitrogen, and carbon) provides a distinct economic advantage over platinum-based catalysts. Since this initial discovery, a significant amount of work has been done towards optimizing the activity and stability of these materials, and it was soon determined that pyrolyzing these materials can yield initial ORR activities comparable to those of commercial Pt/C catalysts.^13–17^ However, despite this progress in improving activity, stability has remained a challenge.^18–24^ Due to the heterogeneous nature of pyrolyzed Fe–N–C sites, fundamental questions regarding the elementary steps in the ORR mechanism and the mechanism of catalyst degradation remain.^18,25–27^

It is generally accepted that Fe–N_4_ sites embedded within the microporous carbon matrix are the primary active sites during the ORR under acidic conditions.^28–30,30–35^ Although these sites can exist as so-called pyrrolic Fe–N_4_ or pyridinic Fe–N_4_ sites, there are typically other sites such as iron oxides, iron nanoparticles, N-doped carbons, and other functional groups present in the pyrolyzed materials.^36–38,38–40^ Recent investigations into the degradation of these materials have proposed three likely mechanisms: (1) the reversible electrochemical oxidation of the carbon support leading to instability of the catalytically active sites, (2) the direct demetallation of the Fe–N_*x*_ active sites through oxidation of iron, and (3) the irreversible chemical oxidation of the carbon support by reactive oxygen species (ROS) such as H_2_O_2_ and OH˙.^18,20,41–44^ For example, H_2_O_2_ can be produced during the 2-electron ORR pathway (eqn (1)) instead of the more desirable 4-electron ORR pathway (eqn (2)). In particular, oxidation of the carbon surface near the active sites has been shown to lead to a modified environment that can impact the binding energy of O_2_ and other intermediates and could also contribute to increased demetallation.^22,42,45–48^

1 O_2_ + 2H^+^ + 2e^−^ → H_2_O_2_

2 O_2_ + 4H^+^ + 4e^−^ → 2H_2_O

Hydroxyl radicals in particular have been increasingly reported in the literature as key species in the chemical oxidation and degradation of Fe–N–C catalysts.^42,44,47,49–51^ ROS are thought to be generated either as direct intermediates during the ORR (eqn (3)) or as byproducts of Fenton-like reactions (eqn (4)) between the iron from the catalyst and H_2_O_2_ generated *via*eqn (1). However, the exact mechanisms for ROS generation are still debated.^49,52–54^

3 O_2_ + 2H^+^ + 2e^−^ → 2OH˙

4 H_2_O_2_ + Fe^2+^ → 2OH˙

Although H_2_O_2_ is frequently observed and quantified using rotating ring disk electrodes (RRDEs),^52,55–57^ radical species have only been observed in a handful of experiments using spectroscopic probe techniques.^43,44,49,58^ However, detecting and quantifying these short-lived, highly reactive species *in situ* with high temporal and spatial resolution is still very challenging.

**Scheme 1:**
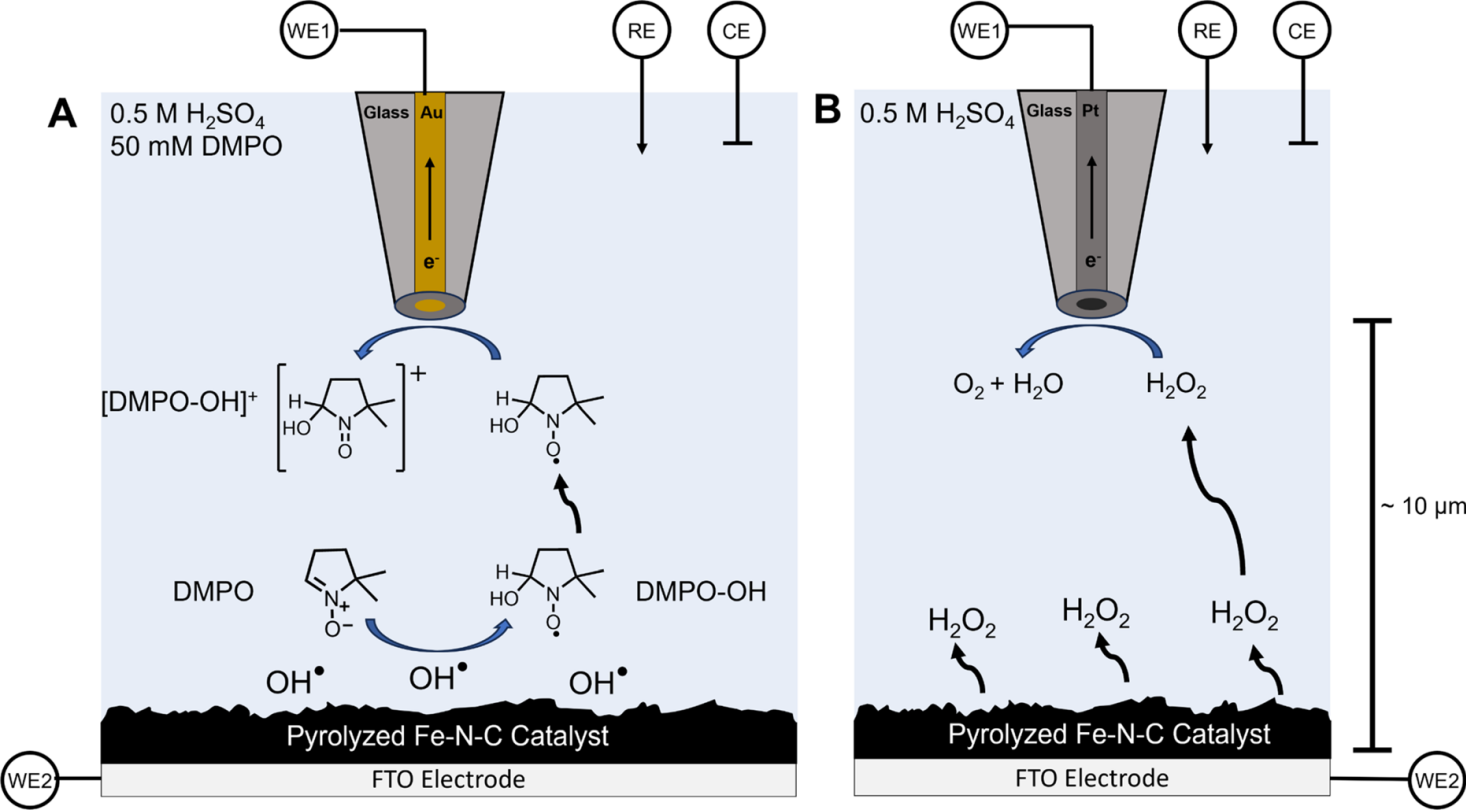
SECM substrate generation–tip collection (SG/TC) for OH˙ detection using DMPO and for H_2_O_2_ detection. (A) Schematic depiction of substrate-generated OH˙ detection using the DMPO spin trap with a 25 μm diameter gold ultramicroelectrode as the tip biased to oxidize the DMPO–OH adduct. (B) A depiction of the SECM H_2_O_2_ detection experiment using a platinum UME biased to perform H_2_O_2_ oxidation.

In this work, we report the use of a scanning electrochemical microscopy (SECM) technique that our group has recently developed for the detection and quantification of transient radical species in real time.^59,60^ SECM is an electrochemical scanning probe technique that positions an ultramicroelectrode (UME) tip over a substrate to achieve high spatial and temporal resolution and to collect substrate-generated species near the source.^61,62^ These attributes are excellent for the detection and quantification of short-lived reactive intermediates and radical species.^63–66^ By adding the commonly used radical spin trap 5,5-dimethyl-1-pyrroline *N*-oxide (DMPO) to solution, a stable, redox-active adduct can form with radical species (DMPO–OH).^67,68^ This adduct can subsequently be detected by the UME in the substrate-generation/tip-collection (SG/TC) mode of SECM (Scheme 1A).^69^ Similarly, this SG/TC configuration can be used to detect hydrogen peroxide if a platinum UME is used to catalytically oxidize H_2_O_2_, similar to the use of a platinum ring in the RRDE (Scheme 1B).^60,70–72^ Here we use these SECM techniques to comprehensively investigate the generation of ROS by a commercial pyrolyzed Fe–N–C catalyst (Pajarito Powder) as well as several model systems, such as iron phthalocyanine, which have been reported to be superior ORR electrocatalysts.^73–75^ We demonstrate the ability to measure radical formation as a result of the ORR process in different amounts at these catalytic surfaces. Our results are supported and confirmed using *ex situ* spectroscopic techniques such as electron spin resonance (ESR) spectroscopy and vibrational spectroscopy.

## 2. Experimental

### 2.1. Chemicals and materials

5,5-Dimethyl-1-pyrroline *N*-oxide (DMPO, 97%, Sigma-Aldrich), hydrogen peroxide (H_2_O_2_ 30%, Fisher), sulfuric acid (H_2_SO_4_ 98%, trace metal grade, Fisher), PMF-011904 Fe–N–C catalyst (Lot 0715, Pajarito Powder), Nafion (5 wt%, Sigma-Aldrich), ethanol (>99.5%, Sigma-Aldrich), Fe(ii) phthalocyanine (Fischer), phthalocyanine (Fischer), 20–30 nm Fe_3_O_4_ nanoparticles (98%, SkySpring Nanomaterials Inc.), Milli-Q DI water (18.6 ohms) were all used without further modification or purification. Fluorine-doped tin oxide (FTO) slides (Delta Technologies, 6–9 ohms, 5% haze) and glassy carbon slides (3 mm thick, Fischer) were used as the substrates.

### 2.2. Substrate preparation

A 4 mg mL^−1^ catalyst ink was prepared by adding 80 mg of Pajarito Powder to 10 mL of water and 10 mL of ethanol along with 100 μL of 5 wt% Nafion. This solution was sonicated for at least 30 min to 1 hour before casting onto a FTO slide. The FTO slides were oxygen plasma cleaned in air for 5 min before casting with the catalyst ink. The substrates were then left in a 100% humidity box overnight after casting to facilitate even film formation and reduce aggregation. The substrates were transferred to a vacuum desiccator for drying and storage. Before SECM experiments, the substrates were dried under a gentle argon stream to remove loose catalysts and large aggregates that may detach in solution. The model catalyst systems were all prepared using an identical procedure (4 mg mL^−1^ inks) except for casting onto a glassy carbon substrate.

### 2.3. Tip preparation

SECM tips were fabricated by sealing a gold (25 μm, 99.999% purity, Goodfellow) or platinum (25 μm, 99.999% purity, Goodfellow) wire inside a quartz glass capillary following previously reported methods.^5,59,76^ Sealing was accomplished with a Narishige PE-2 pipette heater/puller. Conductive carbon paint (Electrobond Resin 61, ConductiveX) was used to create a connection from the gold or platinum wire sealed in the glass to a silver wire. The metal tips were exposed and sharpened to an *R*_g_ < 5 using sandpaper. The SECM tips were polished with 0.05 μm alumina slurry on polishing pads (Buehler) before rinsing and sonicating in Milli-Q deionized water before each experiment.

### 2.4. Scanning electrochemical microscopy

SECM experiments were performed using a 920D CHI scanning electrochemical microscope. Ultramicroelectrodes (UMEs) of either 25 μm diameter gold or platinum were used as the probes. The counter electrode was a graphite rod. The reference electrode was a homemade Ag/AgCl (3 M KCl) reference with a Vycor frit. This reference was separated from the working solution using a 0.1 M NaClO_4_ agar salt bridge. All electrochemical potentials are against this reference unless otherwise indicated. The FTO or glassy carbon substrate electrodes used copper tape to create a contact, but the solution was isolated from the copper connection with a Teflon cell with a Viton O-ring exposing the desired electrode area. The probes were positioned using O_2_ as a redox mediator for a negative approach curve to avoid the use of other mediators resulting in contamination of the solution. Substrate Generation/Tip Collection (SG/TC) experiments were performed after the tip was approached to the surface at approximately 10 μm away. For both the DMPO–OH and H_2_O_2_ collection, the gold or platinum UME, respectively, was biased to 1 V. All voltammograms were recorded at a scan rate of 10 mV s^−1^ unless otherwise specified. Accelerated stress tests (ASTs) were performed with chronoamperometric pulses between 1 V and 0 V at one-second intervals. Each cycle consisted of 320 pulses (Fig. S9†). The Teflon SECM cells were custom machined and cleaned with piranha solution (3 : 1 H_2_SO_4_ : H_2_O_2_), and triple washed and sonicated in Milli-Q water before each use.

### 2.5. Electron spin resonance (ESR) measurements

All ESR measurements were performed with a radical spin trap in an aqueous solution. The ESR experiments were performed by aliquoting the solution into a Wilmad Glass quartz flat cell (Suprasil TM110). The spectra were acquired at room temperature (∼298 K) with a Bruker EMXPlus X-band instrument. Measurement conditions were 3480 G center field, 8 scans, 20 mW microwave power, 2.0 × 10^3^ gain, 1 G modulation amplitude, 100 kHz modulation frequency, 0.064 second time constant, and a microwave frequency of ∼9.7775 GHz. ESR fitting was performed using the EasySpin Matlab program.^77^ Additional fitting parameters and details are provided in the text.

### 2.6. Raman spectroscopy

Raman spectroscopy was performed *ex situ* using a confocal Nanophoton Raman 11 microscope. Each spectrum was acquired using a 532 nm excitation laser at ∼2.5 mW power with a LU Plan Fluor ×50/NA 0.8 objective. The grating was 600 g mm^−1^ with a center wavelength of 1800 cm^−1^ and a slit width of 50 μm. Each spectrum was acquired with an integration time of 5 seconds and 5 averages.

### 2.7. Catalyst characterization

The commercial Pajarito Powder catalyst was additionally characterized using X-ray photoelectron spectroscopy (XPS), scanning electrochemical microscopy (SEM) with energy dispersive X-ray (EDX) spectroscopy, nitrogen adsorption isotherms, and elemental analysis. Experimental details and catalyst characterization using the above techniques are provided in the ESI in Sections S1 and S2 (Fig. S1–S6).†

## 3. Results and discussion

### 3.1. SECM measurements of radical and H_2_O_2_ production on pyrolyzed Fe–N–C

**Fig. 1:**
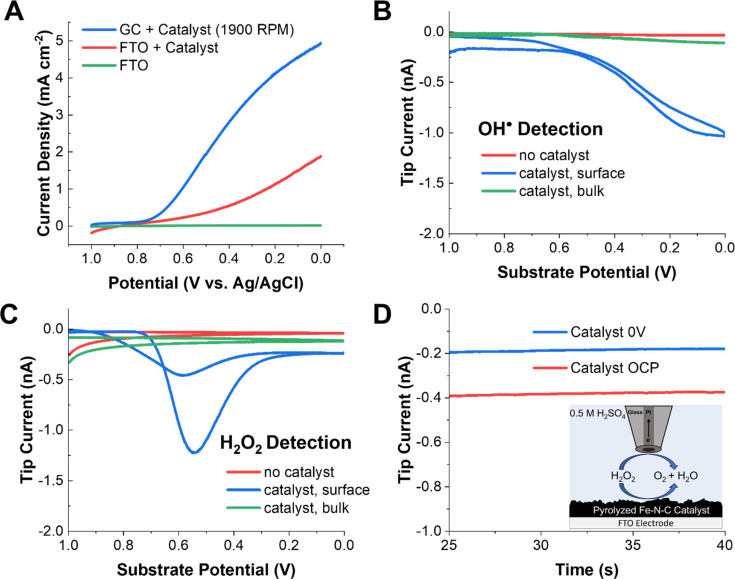
SECM results for radical and H_2_O_2_ detection on pyrolyzed Fe–N–C catalysts. (A) Substrate LSVs of the catalyst on a rotating GC electrode and a stationary FTO substrate in oxygen-saturated 0.5 M H_2_SO_4_, as well as a catalyst-free FTO blank. (B) The radical SG/TC response at the gold tip as the substrate potential is swept at 10 mV s^−1^ with 25 mM DMPO in solution. (C) The H_2_O_2_ SG/TC response on a Pt tip as the substrate potential is swept at 10 mV s^−1^. (D) Redox competition mode SECM with a Pt tip located above the Fe–N–C in 0.5 mM H_2_O_2_ solution in 0.5 M H_2_SO_4_ purged with argon, with the substrate biased to the ORR and under open circuit conditions.

Fig. 1A shows the typical linear sweep voltammetry (LSV) profile of the commercial Pajarito catalyst (4 mg mL^−1^) on an FTO substrate (used for SECM) and on a GC substrate in 0.5 M H_2_SO_4_ under oxygen-saturated conditions. The measurement on GC was done to verify that the activity of the catalyst ink was comparable to that reported in the literature using a rotating disk electrode. The onset of oxygen reduction was clearly observed at both FTO and GC at ∼0.7 V *vs.* 3 M Ag/AgCl. Furthermore, the limiting current observed for the rotated GC electrode is consistent with that reported elsewhere.^47,49,55^ In the absence of the catalyst, no redox activity was observed at the substrate.

To detect radical species, DMPO was added to the acidic electrolyte. DMPO is a common radical spin trap used to extend the lifetime of oxygen-based radicals. Our group has recently shown that when DMPO reacts with OH˙, a redox-active adduct is formed that can be electrochemically detected.^59^ To probe whether radicals are generated by Fe–N–C catalysts performing the ORR, a gold UME tip was approached to the substrate (Fig. S7†) at a distance of ∼10 μm and 25 mM DMPO was added to the solution (Scheme 1A). In this SG/TC configuration, a redox-active species generated at the substrate (*e.g.*, DMPO–OH, only if OH˙ is produced) can be collected by the tip electrode, in an experiment conceptually analogous to an RRDE measurement. However, the SECM configuration offers superior temporal resolution and collection efficiency for the detection of dilute and short-lived species.

In the DMPO–OH collection experiment, CV was performed at the substrate while the gold tip was biased at 1 V to detect DMPO–OH. The use of a gold UME is important to ensure that the current response is not convoluted with that from hydrogen peroxide, which can be electrocatalytically oxidized at the same potentials at a platinum tip (Fig. S12†). Under these conditions, we observed a collection current at the tip which closely followed the ORR activity at the substrate. This shows that radical species, presumably OH˙, are being generated in the vicinity of the substrate electrode. Additionally, minimal tip current is observed in either the absence of a catalyst on the electrode surface or if the tip is removed into the bulk solution (>500 μm). This further demonstrates that the collection signal originates from species generated during the ORR at the Fe–N–C catalyst. This strongly suggests that the radicals are produced as byproducts or intermediates of redox processes occurring during the ORR at the Fe–N–C catalyst.

A common experiment performed in the ORR literature is the collection of H_2_O_2_ produced by the unwanted two-electron pathway with a RRDE. An analogous SECM SG/TC experiment was performed using a platinum tip biased to 1 V to detect H_2_O_2_ produced by the substrate (Fig. 1C). In this case, a significantly different potential dependent behavior was observed for H_2_O_2_, with a maximum in the measured tip current observed at ∼0.55 V. This behavior has been observed previously in RRDE experiments using high catalyst loadings (>2 mg cm^−1−2^).^55^ This is because the catalyst has been reported to reduce hydrogen peroxide at higher overpotentials (eqn (5)).

5 H_2_O_2_ + 2H^+^ + 2e^−^ → 2H_2_O

To probe this possibility, 0.5 mM H_2_O_2_ was added to the solution, and the solution was purged with argon. When the platinum tip was biased to 1 V in this H_2_O_2_-containing solution while the catalyst was kept at open circuit, a steady state current of ∼0.4 nA was observed due to H_2_O_2_ electrolysis at the tip. However, when the catalyst was biased to reducing potentials, the current at the tip decreased by ∼50% to 0.2 nA. This redox competition behavior arises because the substrate consumes H_2_O_2_ in the vicinity of the tip, thus lowering the tip current and demonstrating that eqn (5) is operative at the substrate.

### 3.2. Spectroscopic confirmation of radical production

**Fig. 2:**
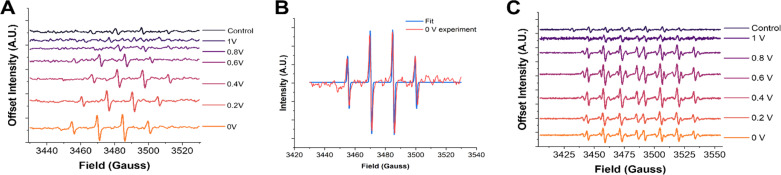
ESR spectra with DMPO and DEPMPO. (A) ESR spectra of 50 mM DMPO after two minutes of electrolysis at the pyrolyzed Fe–N–C catalyst substrate at increasingly reducing potentials. (B) ESR spectra of 50 mM DEPMPO after two minutes of electrolysis at the pyrolyzed Fe–N–C catalyst substrate at increasingly reducing potentials. (C) Fitting of the 0 V DMPO ESR experiment to obtain the hyperfine coupling constants and confirm the radical species present. Simulated spectra for different possible adducts are provided in the ESI.†

We will now elaborate on the detection of radical species. To determine the radical species present and support the SECM experiments, we carried out ESR measurements of the electrolyzed solutions. The solutions were generated by electrolyzing an oxygen-saturated acidic electrolyte containing DMPO at each potential for two minutes. Immediately after the electrolysis, a small aliquot (∼500 μL) of solution was taken from the vicinity of the electrode surface and placed in a vial. The vials were immediately transported to the ESR, and spectra were recorded in a quartz flat cell to minimize the dielectric effects of the aqueous solution. The potential dependent ESR spectra obtained using DMPO as the spin trap qualitatively confirmed hydroxyl radicals to be the predominant radical species present (Fig. 2A). The 1 : 2 : 2 : 1 peak splitting observed is characteristic of the DMPO–OH adduct.^59,68,78^ Although the signal intensities were not calibrated with an internal standard, there is a clear increase in the ESR signal intensity as the potential is made more negative. This agrees with the radical generation trends observed *via* SECM in Fig. 1B. Furthermore, the ESR spectra of DMPO obtained at 0 V were fit using the Easy Spin program (Fig. 2B).^77^ The fitting considered the possible contributions from DMPO–OH and DMPO–OOH, the two most likely radical adducts produced.^79^ It was found that the best fit was achieved when DMPO–OH was the sole adduct present with hyperfine splitting constants of ^14^N = 41.6657 MHz and ^1^H = 41.7680 MHz, in good agreement with previous literature on DMPO–OH.^67,78^

However, as has been noticed in previous work, other oxygen centered radicals trapped by DMPO, such as OOH˙ and O_2_˙^−^, can rapidly decay into the DMPO–OH adduct.^68,79–81^ Although the half-life of these other species may make these adducts accessible on the time scale of the electrochemical experiments (seconds), they may not be apparent when performing ESR experiments, which can take several minutes. To ensure that hydroxyl radicals really are the predominant radical species generated, another DMPO analogue spin trap was used known as 5-(diethoxy phosphoryl)-5-methyl-1-pyrroline-*N*-oxide (DEPMPO).^81,82^ This spin trap is known to stabilize OOH˙ and O_2_˙^−^ radicals longer than DMPO. The same two-minute constant potential electrolysis procedure followed by ESR was repeated using DEPMPO (Fig. 2C). In these results, a more complex 1 : 2 : 2 : 1 : 1 : 2 : 2 : 1 pattern is observed. This additional splitting is due to the additional magnetic nucleus (^31^P) in the DEPMPO structure. Additionally, other minor peak splitting's are observed in the ESR spectrum, most notably in the 0.6 V spectra. These minor peaks are likely from the DEPMPO–OOH adduct, as shown in the ESR simulation of this structure in the ESI (Fig. S8).† The contribution of these minor peaks to the spectrum was largest at ∼0.6 V, which coincides with the peak of H_2_O_2_ production measured *via* SECM. However, a good fit and quantitative ratio of the abundance of the two radical species could not be accurately obtained due to the difficulty in fitting the DEPMPO–OOH structure because of the variety of conformations and isomers possible.^83,84^ Altogether, these observations strongly suggest that while the presence of OOH˙ cannot be discarded, and possibly correlated to the formation of H_2_O_2_, OH˙ is by far the most abundant radical detected.

### 3.3. OH˙ and H_2_O_2_ radical formation trends upon Fe–N–C degradation

We now turn to understanding how radical production changes as the catalyst degrades. It has been hypothesized that leaching of the iron from the FeN_4_ active sites increases the radical production due to the precipitation of radical-forming iron oxide nanoparticles, or due to increased Fenton-type reactions (eqn (4)).^34,38^ To perform these measurements, radical and H_2_O_2_ generation were monitored using the same SG/TC experiment after a square wave potential waveform was applied to the substrate. The substrate was pulsed between 1 V and 0 V at 1-second intervals. Each accelerated stress test (AST) cycle consisted of 320 pulses (Fig. S9†). After each AST cycle, the substrate was allowed to rest for 5 minutes before an SG/TC experiment was initiated. This process was repeated nine times for each substrate. This AST procedure was chosen since it showed a high rate of catalyst degradation in a short time, a key factor considering the increasing effects of solution evaporation and spin trap decomposition during longer SECM experiments.

**Fig. 3:**
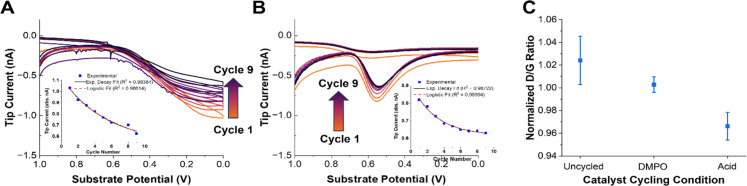
Accelerated stress test (AST) experiments. (A) OH˙ SG/TC detection using 50 mM DMPO as the catalyst is degraded using AST. The arrows indicate a decrease in the current going from cycle 1 to 9. The inset shows the tip current at 0 V as a function of cycle number. The solid line shows an exponential decay fit and the dashed line shows a logistic fit. (B) The H_2_O_2_ SG/TC response as the catalyst is degraded. The arrows indicate a decrease in the current going from cycle 1 to 9. The inset shows the tip current at 0.55 V (peak current) as a function of cycle number. The solid line shows an exponential decay fit and the dashed line shows a logistic fit. (C) The normalized D/G peak intensity ratio from the Raman spectra of pyrolyzed Fe–N–C catalysts degraded in different solutions (with or without DMPO). Representative Raman spectra are shown in Fig. S10.†

The SG/TC CVs for both the radicals (Fig. 3A) and H_2_O_2_ (Fig. 3B) showed a decrease in tip current as a function of catalyst degradation. Plotting the tip current at the potential of maximum collection for each cycle (0 V for DMPO, 0.55 V for H_2_O_2_) showed an exponential or logistic decay in ROS generated (Fig. 3A and B, insets). This type of decay has been shown by kinetic modeling to probably correspond to active site loss, possibly through an autocatalytic mechanism.^85^ This would suggest that the generation of ROS by Fe–N–Cs is closely linked to the catalytic activity, and could be indicative of irreversible oxidative degradation of the catalyst. If a purely chemical Fenton reaction were the dominant mechanism in the production of radical species, an increasing trend would be expected as the catalyst degrades (eqn (4)). It has been shown that degradation induces demetallation and the increase of free Fe^2+^ ions in solution, however, based on our results this does not seem to accelerate the rate of degradation.^36^ We confirmed this trend by comparing the amount of iron dissolved in the electrolyte after cycling the catalyst under different conditions (Fig. S14†). Although the standard deviation for the AST measurements was large, the iron dissolution results suggest that an increase in radical production would be expected if a chemical Fenton mechanism was the dominant source of hydroxyl radicals. An electro-Fenton mechanism or chemical oxidation by desorbed radical intermediates could be an explanation for the inverse correlation between initial catalytic activity and catalyst stability often observed across a variety of Fe–N–C systems.^18^

Finally, we note that the decrease in the measured tip currents after the ASTs for both OH˙ and H_2_O_2_ was estimated at 14% and 23%, respectively. This qualitative loss of activity was obtained by comparing the maximum tip current (0 V for OH˙ and 0.55 V for H_2_O_2_) before and after AST cycling of the catalyst. The tip current after the AST for the OH˙ was taken from the values derived after the DMPO solution was replaced (Fig. S13†). The only difference between these experiments was the presence of a significant concentration of DMPO in solution as a radical scavenger. Given the smaller changes in activity in the presence of DMPO, we speculated that the catalyst stability may be improved in the presence of a radical scavenger.^51,86^ Despite the fact that OH˙ and H_2_O_2_ production may not be directly correlated with catalyst activity, the reduced production of both species after cycling further emphasizes the likely role of ROS in the degradation mechanism in operating Fe–N–Cs.

To qualitatively confirm whether the presence of DMPO as a radical scavenger could improve the catalyst stability, Raman spectra of the catalyst were taken before and after the ASTs under different conditions. Raman spectroscopy has been shown to provide an effective means of observing the extent of carbon corrosion in Fe–N–C catalysts.^24,87^ The ratio between the D or D^3^ and G peaks can be informative as to the loss of “defects” (*i.e.*, active sites) present in the material.^24,28^ Recording Raman spectra with a 532 nm laser with a ×50 confocal objective gave representative spectra shown in Fig. S10.† The data points in Fig. 3C are an average of three normalized spectra. The D/G peak ratio between the fresh (uncycled) catalyst and the catalyst degraded in the presence or absence of DMPO shows that there is a more significant reduction in the D peak intensity in the absence of DMPO (Fig. 3C). This qualitatively supports the previous observations that radical species are involved in the chemical oxidation of Fe–N–C catalysts and the loss of active sites, and that radical scavenging could improve catalyst stability.

However, understanding the exact nature of the chemical oxidation of the Fe–N–C catalyst requires further *in situ* measurements and simultaneous structural correlations. These analyses are made challenging by the large variety of functional groups and active sites present in pyrolyzed materials.

### 3.4. Comparison of OH˙ generation on active site model systems

The pyrolyzed Fe–N–C catalysts are heterogeneous, porous materials with a variety of active sites including graphitic carbon, N-doped carbon, pyrrolic and pyridinic Fe–N_4_ sites, and iron oxide nanoparticles. Thus, it is challenging to understand which structural features are contributing towards the production of ROS. It is important to understand the relative contribution to radical production for each active site under operating conditions so that rational synthetic approaches can be pursued to improve catalyst stability. For example, it has been proposed in previous work that iron oxides or iron nanoparticles are a significant source of radicals and that pyrrolic Fe–N_4_ active sites are less durable than pyridinic ones.^39,88,89^ Commercially available model systems were obtained to explore OH˙ radical formation on these types of materials. Phthalocyanine (H_2_Pc), iron(ii) phthalocyanine (FePc), and 20 nm Fe_3_O_4_ iron oxide nanoparticles along with bare glassy carbon were explored. Phthalocyanines are frequently used as a molecular model system for the pyrrolic M–N_4_ sites present in most Fe–N–C catalysts. H_2_Pc could model either demetallation of Fe–N_4_ sites or metal-free nitrogen-doped carbon sites. Additionally, iron and iron oxide nanoparticle phases are frequently present as unwanted impurities from the pyrolysis procedure in the synthesis of most Fe–N–C catalysts and may form *in situ* from leached iron ions as the catalyst degrades.

**Fig. 4:**
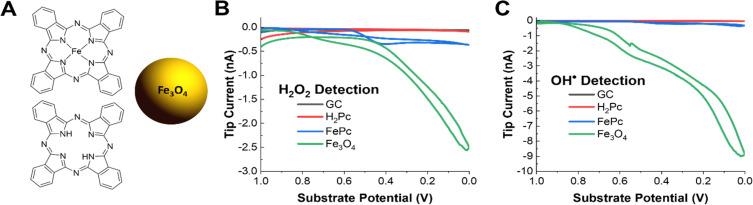
SECM measurements of OH˙ and H_2_O_2_ generation on active site model systems. (A) The structure of iron(ii) phthalocyanine, phthalocyanine, and schematic of the 20 nm Fe_3_O_4_ nanoparticle. (B) H_2_O_2_ SG/TC response of each model catalyst system on a GC electrode in oxygen-saturated 0.5 M H_2_SO_4_. (C) OH˙ SG/TC detection using 50 mM DMPO of each model catalyst system on a GC electrode in oxygen saturated 0.5 M H_2_SO_4_.

Performing the same radical and H_2_O_2_ SG/TC SECM experiments showed that only FePc and Fe_3_O_4_ – the Fe-containing species in our selection of materials – could generate detectable amounts of H_2_O_2_ or radicals under ORR conditions (Fig. 4B and C). This reinforces the nature of metal-centered ORR activity and the possible role of iron ions in radical-generating reactions. Additionally, although these experiments were performed at nominally the same catalyst loadings (4 mg mL^−1^), Fe_3_O_4_ produced significantly more ROS as measured by the tip currents. As done before, we used ESR to verify that the OH˙ radical was the main species detected (Fig. S11†). It is also important to note that although radical and H_2_O_2_ production by Fe_3_O_4_ is overwhelmingly higher than that of FePc, the latter still produced these species at comparable tip currents to the pyrolyzed system. This suggests that although the removal of unwanted iron nanoparticle phases is an important step to improving the stability of pyrolyzed Fe–N–C catalysts, even the desired Fe–N_4_ active sites could be generating ROS that could contribute to the degradation of the catalyst. It has been reported in the literature that pyridinic Fe–N_4_ sites are more stable than pyrrolic sites, so further investigations using our technique could help support observations addressing the relative stability differences observed between these two sites in pyrolyzed systems as well.^39^

## 4. Conclusions

We have investigated ROS production by an Fe–N–C catalyst during the ORR in real-time using a novel scanning electrochemical microscopy methodology *via* a redox-active DMPO spin trap for detecting the OH˙ radical, as well as H_2_O_2_ collection experiments. SECM reported on very different trends for the production of these two species, with OH˙ production increasing monotonically with ORR activity and H_2_O_2_ production peaking at ∼0.55 V *vs.* Ag/AgCl. We confirmed that OH˙ was the main radical species detected using *ex situ* ESR with both DMPO and DEPMPO spin traps. Aggressive accelerated stress testing of the catalyst while monitoring for OH˙ and H_2_O_2_ generation also showed a decreasing production as the catalyst degrades. Additionally, the presence of spin traps as radical scavengers in solution led to a decrease in the degradation of the catalyst, confirming previous reports implicating hydroxyl radicals in the deactivation of Fe–N–C catalysts. Finally, the structural origin of radical production was investigated using model systems for the possible active sites. Iron phthalocyanine (FePc, a model for pyrrolic Fe–N_4_) and 20 nm Fe_3_O_4_ nanoparticles both showed the ability to generate ROS. Importantly, SECM measurements of OH˙ production indicated that Fe_3_O_4_ is a major producer of these species, while FePc generates this species in a comparable manner to the pyrolyzed material. In summary, SECM was used to provide direct evidence for the proposed degradation pathways of Fe–N–C ORR catalysts by measuring hard-to-observe radical intermediates and byproducts in real time. We posit that our SECM technique will be useful to determine mechanistic differences across a variety of electrocatalytic materials for the ORR and other reactions.

## Data availability

All the data supporting this article have been uploaded as part of the ESI.†

## Author contributions

Seth T. Putnam (conceptualization, methodology, validation, formal analysis, investigation, writing – original draft, writing – review & editing, visualization) and Joaquín Rodríguez-López (conceptualization, writing – review and editing, supervision, resource, funding acquisition).

## Conflicts of interest

There are no conflicts to declare.

## Supplementary Material

SC-015-D4SC01553C-s001
