# Supplementary material for: Real-time investigation of reactive oxygen species and radicals evolved from operating Fe–N–C electrocatalysts during the ORR: potential dependence, impact on degradation, and structural comparisons
Source: Chem Sci. 2024 May 31;15(26):10036–45. doi: 10.1039/d4sc01553c (PMC11220586; doi:10.1039/d4sc01553c)
Supplement: SC-015-D4SC01553C-s001 [file SC-015-D4SC01553C-s001.pdf]

Electronic Supplementary Material (ESI) for Chemical Science.
This journal is © The Royal Society of Chemistry 2024

Real-time investigation of reactive oxygen species and radicals evolved from operating Fe-N-C electrocatalysts during the ORR: potential dependence, impact on degradation, and structural comparisons

Seth Putnam[1], Joaquín Rodríguez-López[1, *]

University of Illinois Urbana-Champaign, Department of Chemistry, 600 Matthews Ave, Urbana, IL 61801.

Contents

Section S1 – Experimental details 2
- X-ray Photoelectron Spectroscopy (XPS) 2
- Scanning Electron Microscopy (SEM) with Energy Dispersive X-ray spectroscopy (EDX) 2
- Elemental Analysis 2
- Nitrogen Adsorption Isotherms 2

Section S2 – Pyrolyzed Fe-N-C catalyst characterization 3
- Figure S1 - X-ray Photoelectron Spectroscopy. 3
- Figure S2 - SEM images and EDX mapping of uncycled catalyst. 4
- Figure S3 – EDX spectra of the uncycled catalyst. 4
- Figure S4 – SEM images and EDX mapping of degraded catalyst. 5
- Figure S5 – EDX spectra of the degraded catalyst. 5
- Table S1 – Elemental analysis of the elements present in the pyrolyzed Fe-N-C catalyst. The values are the averages of two independent runs. 6
- Figure S6 – A representative nitrogen adsorption isotherm of the Pajarito catalyst. 7

Section S3 – Supplementary experiments 8
- Figure S7 –A representative approach curve. 8
- Figure S8 –Simulated ESR of possible ROS. 9
- Figure S9 – Accelerated stress test waveform and RDE. 10
- Figure S10 – Representative Raman spectra. 11
- Figure S11 – ESR of model systems. 12
- Figure S12 – H_2O_2 at gold and platinum electrodes. 13
- Figure S13 – DMPO solution regeneration after AST. 14
- Figure S14 – Solution iron content (ICP-MS). 15

References 16

Section S1 – Experimental details

X-ray Photoelectron Spectroscopy (XPS)

X-ray photoelectron spectra were collected using a Kratos Axis Supra+ spectrometer using an Al Ka/Ag La dual anode monochromatic X-ray source. The measured binding energies were calibrated to the C=C graphitic carbon peak at 284.5 eV.

Scanning Electron Microscopy (SEM) with Energy Dispersive X-ray spectroscopy (EDX)

Scanning electron microscopy images of the cycled and degraded pyrolyzed catalyst were obtained using a Hitachi S-4800 while energy dispersive X-ray spectra and maps were obtained using an Oxford Ultim Max attachment installed on the same electron microscope.

Elemental Analysis

CHN results were obtained by averaging two runs performed by an Exeter Analytical CE440 CHN analyzer. Iron content was determined by ICP-MS using an NExION 350D instrument. The combustion for the CHN analysis was stopped at 2000 C° to prevent the formation of carbides. Therefore, the carbon, nitrogen, and hydrogen content are likely an underestimation of the true value. However, the overall iron content is in good agreement with the expected value and those obtained using EDX on an uncycled catalyst.

Nitrogen Adsorption Isotherms

Three nitrogen adsorption isotherm runs were recorded on a Micromeritics 3Flex at 77 K. The average surface area was found to be 648.3623 +/- 0.8044 m^2/g. The BJH desorption surface area of pores above 15 angstroms was 395.1759 +/- 2.1600 m^2/g with an average pore diameter of 74.040 +/- 0.497 angstroms. These results confirm the high surface area and microporous nature of the commercial pyrolyzed Fe-N-C catalyst.

Section S2 – Pyrolyzed Fe-N-C catalyst characterization

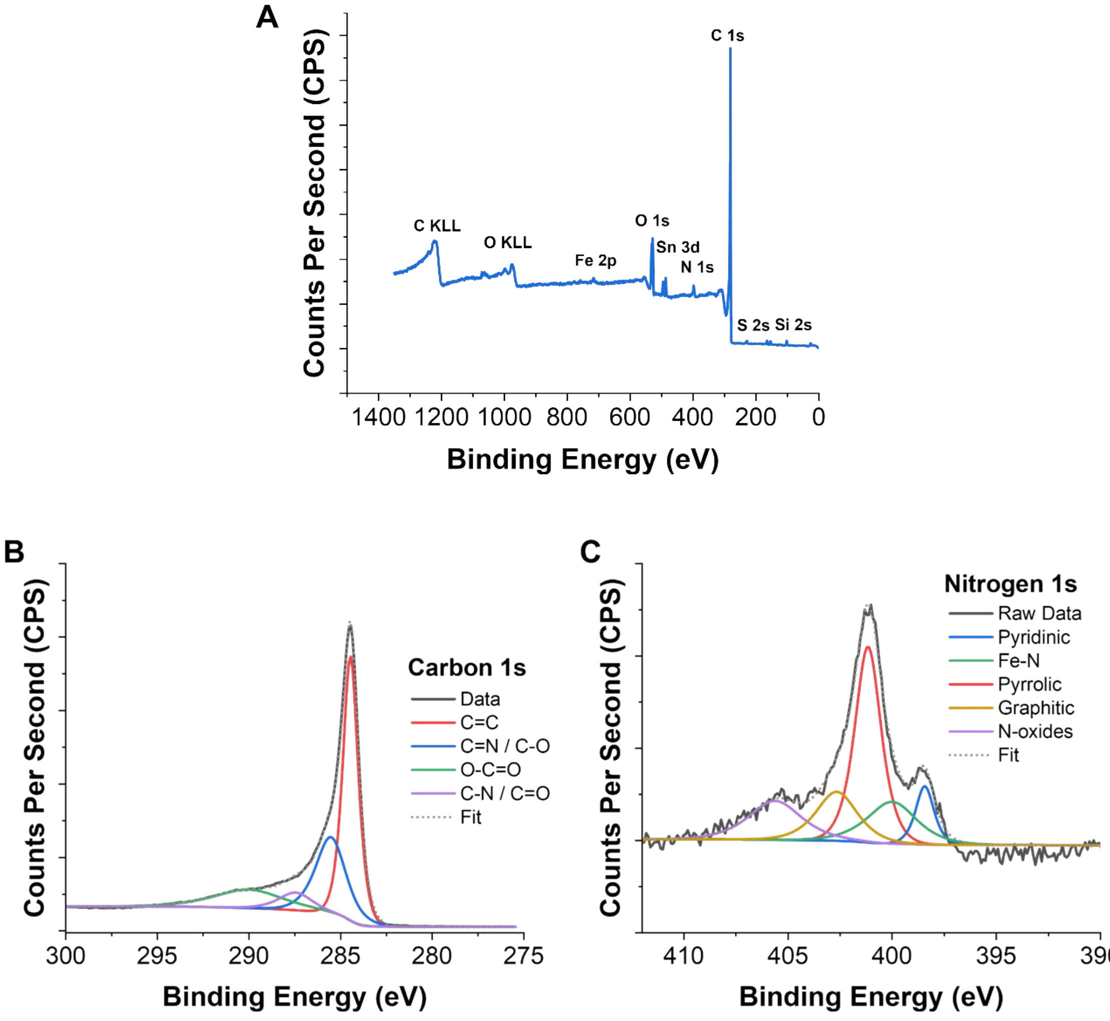

Figure S1 - X-ray Photoelectron Spectroscopy. A) Survey scan of the uncycled pyrolyzed Fe-N-C catalyst deposited on an FTO electrode. **B)** High resolution carbon 1s region showing the variety of carbon bonding motifs present. **C)** high resolution nitrogen 1s region showing the five major peaks (pyridinic, M-N_x, pyrrolic, graphitic, and oxidic). The results are in good agreement with those obtained in previous literature on this catalyst.[1,2]

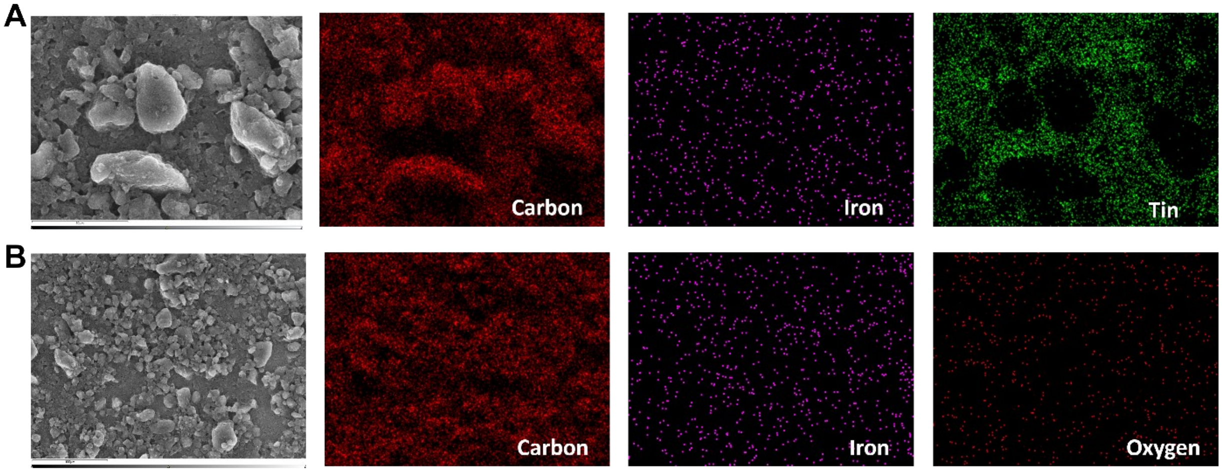

Figure S2 - SEM images and EDX mapping of uncycled catalyst. Images of two selected regions (**A, B**) of the uncycled pyrolyzed catalyst deposited over an FTO substrate. EDX mapping of each image shows the carbon, iron, and tin or oxygen spatial distribution.

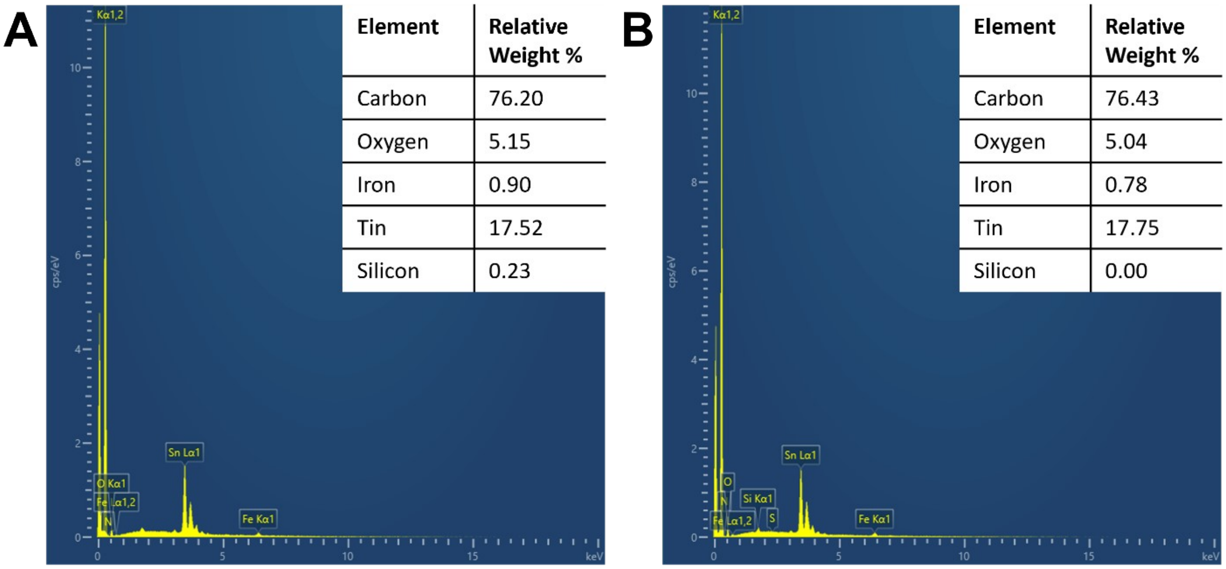

Element	Relative Weight %
Carbon	76.20
Oxygen	5.15
Iron	0.90
Tin	17.52
Silicon	0.23

Element	Relative Weight %
Carbon	76.43
Oxygen	5.04
Iron	0.78
Tin	17.75
Silicon	0.00

Figure S3 – EDX spectra of the uncycled catalyst. EDX spectra of the pyrolyzed Fe-N-C catalyst from **Figure S2 (A, B)**. The relative weight percent of the various detected elements is shown in the inset tables. The FTO substrate contributes to the tin and silicon signals.

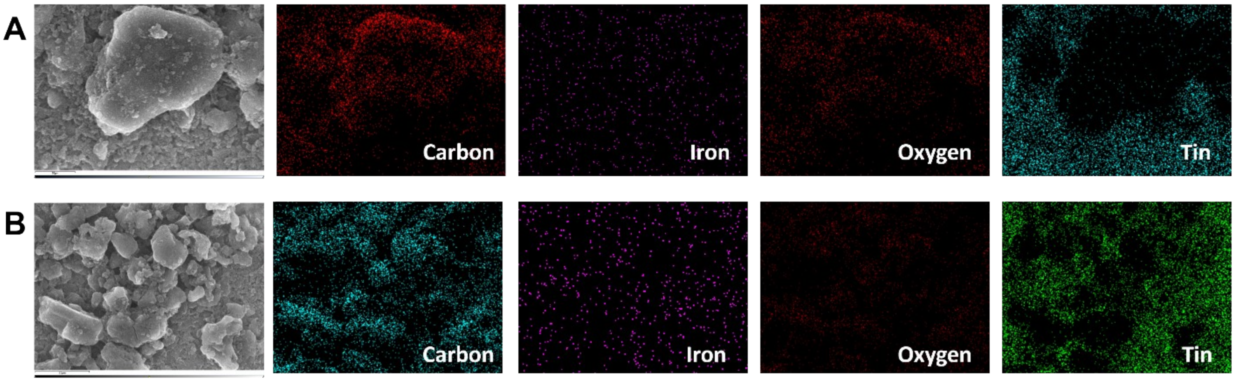

Figure S4 – SEM images and EDX mapping of degraded catalyst. Images of two selected regions (**A, B**) of the pyrolyzed catalyst after cycling according to the accelerated stress test. EDX mapping of each image shows the carbon, iron, oxygen and tin spatial distribution.

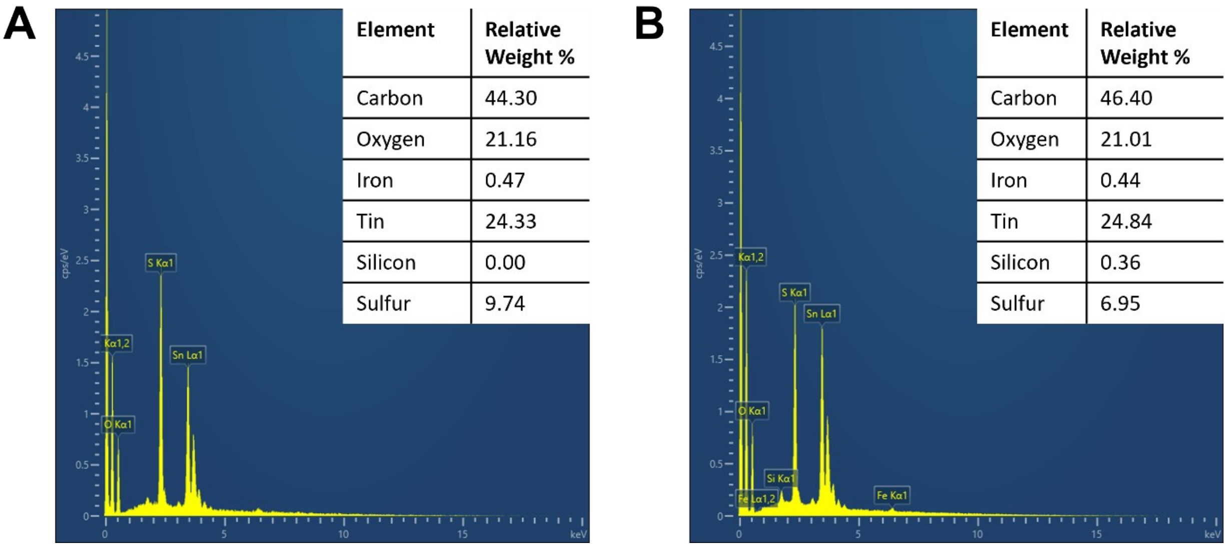

Figure S5 – EDX spectra of the degraded catalyst. EDX spectra of the pyrolyzed Fe-N-C catalyst from **Figure S4 (A, B, C)**. The relative weight percent of the various detected elements is shown in the inset tables. The FTO substrate contributes to the tin and silicon. The increased sulfur and oxygen signals are likely from residual electrolyte (H_2SO_4).

Element	Weight %
C	81.68
H	1.22
N	2.58
Fe	0.947

Table S1 – Elemental analysis of the elements present in the pyrolyzed Fe-N-C catalyst. The values are the averages of two independent runs.

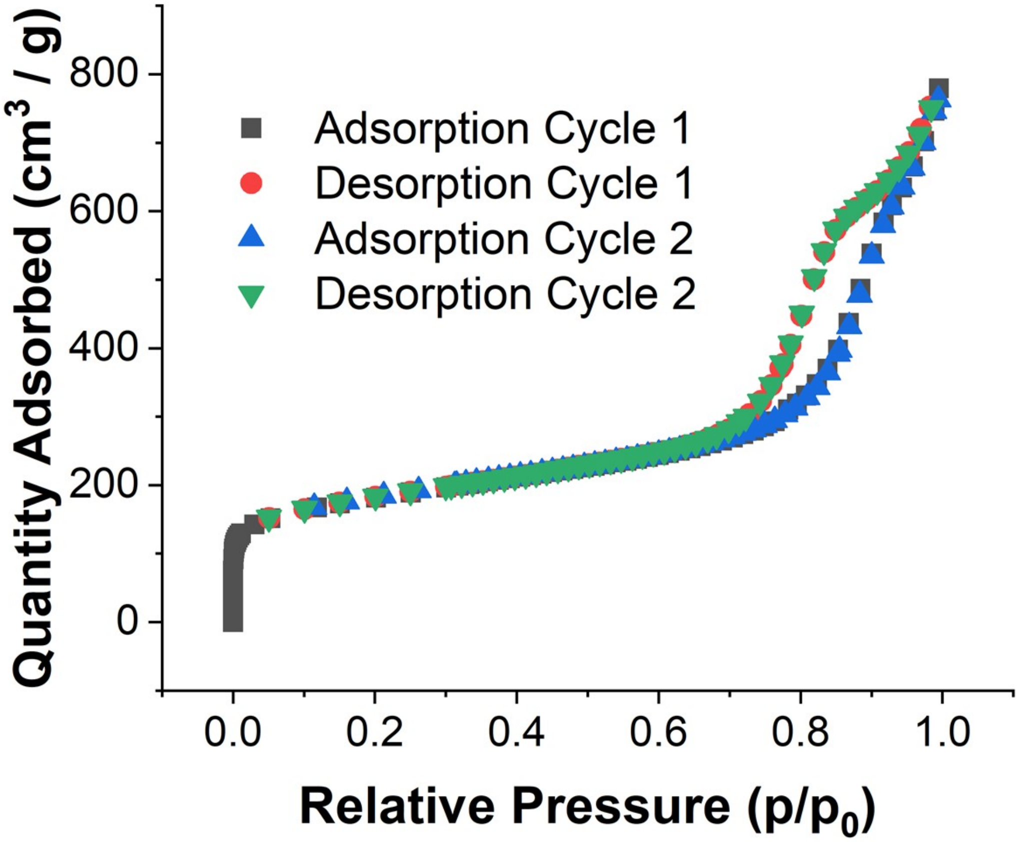

Figure S6 – A representative nitrogen adsorption isotherm of the Pajarito catalyst.
Three independent measurements were performed, and the average surface area was found to be 648.3623 +/- 0.8044 m^2/g.

Section S3 – Supplementary experiments

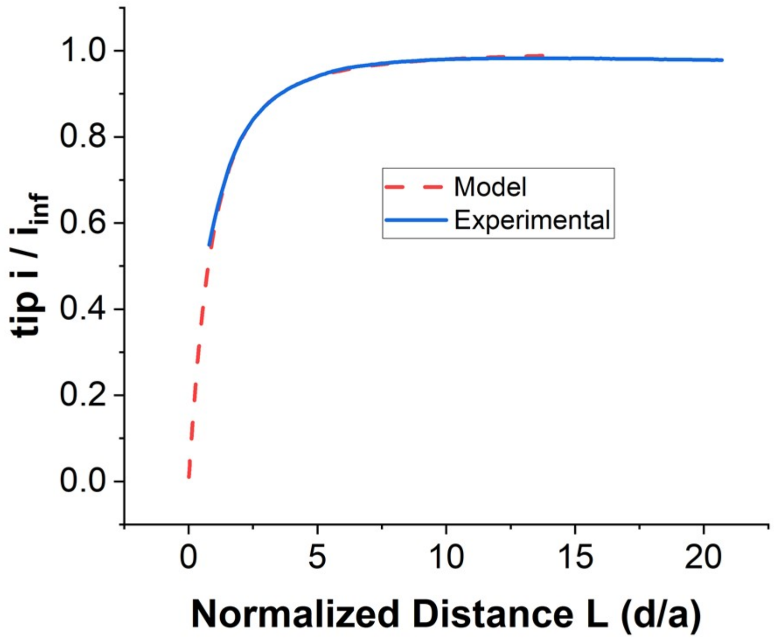

Figure S7 –A representative approach curve. A representative fitted negative feedback approach curve using O_2 as the mediator with the tip held at -0.2V at a 25 µm diameter Au UME which is fit to a model negative feedback curve. The fitting procedure extracts an Rg of 5 and a normalized distance of 0.8 (~10 µm).[3]

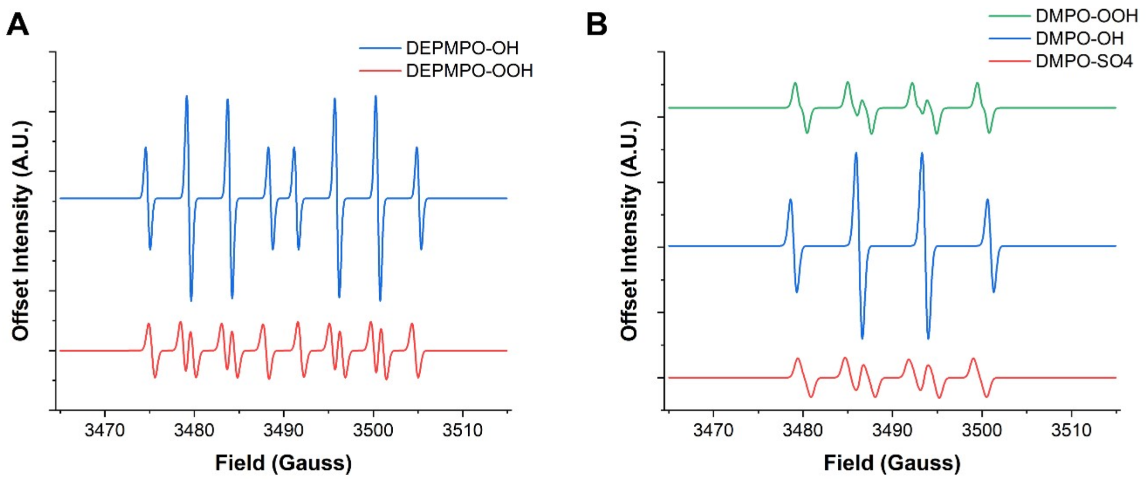

Figure S8 –Simulated ESR of possible ROS. Simulated ESR spectra of **A)** DEPMPO-OH and DEPMPO-OOH. The DEPMPO-OOH spectra were simulated with the parameters: nuclei (^{14}N, ^{31}P, ^{1}H, and ^{1}H) with hyperfine splitting constants of 13.13, 47.61, 13.11, and 0.3 MHz respectively and a linewidth of 0.05 mT and a g-factor of 2.0071. The DEPMPO-OH spectra used similar values but omitted the second ^{1}H. **B)** DMPO-OH, DMPO-OOH, and DMPO-SO_4. The DMPO-OH spectra were simulated with the parameters: nuclei (^{14}N, ^{1}H) with hyperfine splitting constants of 41.6657 and 41.7680 MHz respectively and a linewidth of 0.1 mT and a g-factor of 2.0071. The DMPO-OOH spectra used similar values but added a second ^{1}H.

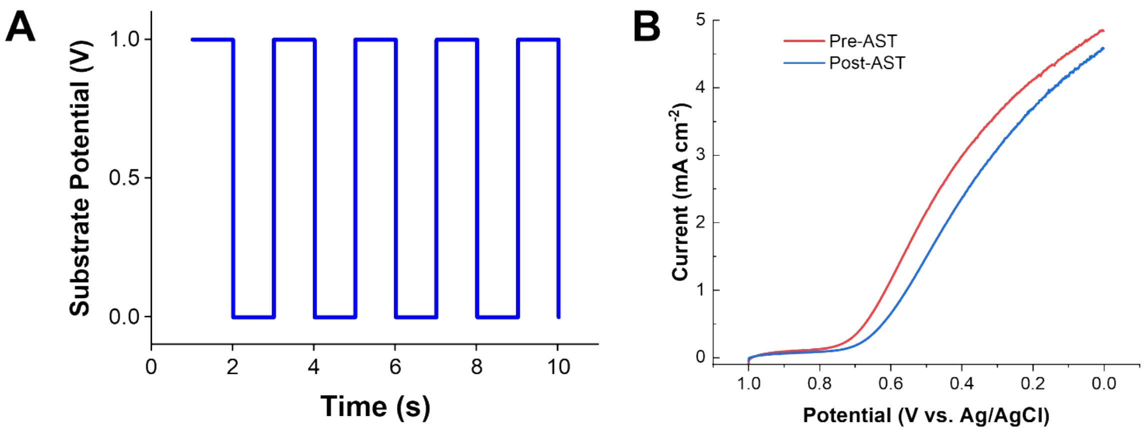

Figure S9 – Accelerated stress test waveform and RDE. A) The accelerated stress test (AST) pulsed potential waveform applied to the catalyst to achieve rapid degradation. Each cycle involved one second pulses between 0 V and 1 V. **B)** RDE LSVs of the pyrolyzed catalyst before and after applying the AST (9 cycles of 320 pulses) in an oxygen saturated 0.5 M H_2SO_4 electrolyte (1900 RPM).

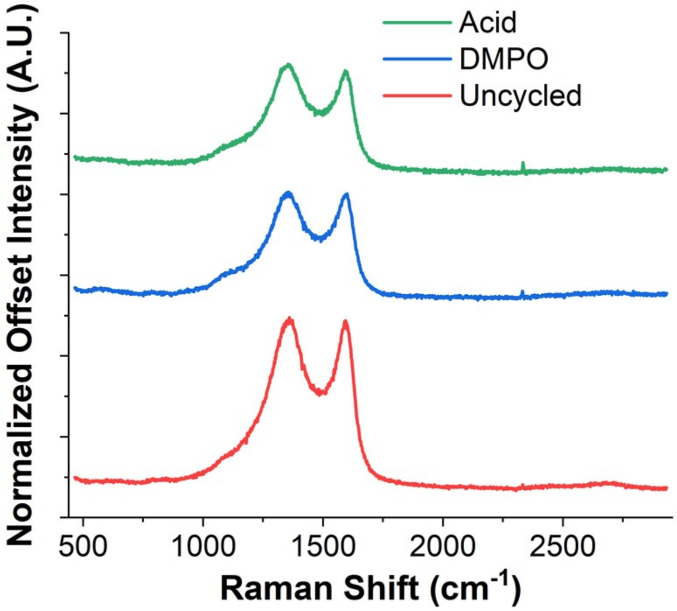

Figure S10 – Representative Raman spectra. Representative Raman spectra of the pyrolyzed Fe-N-C catalyst taken ex situ after degradation using the AST procedure described using different solution conditions. The average D/G ratios are presented in Figure 3.

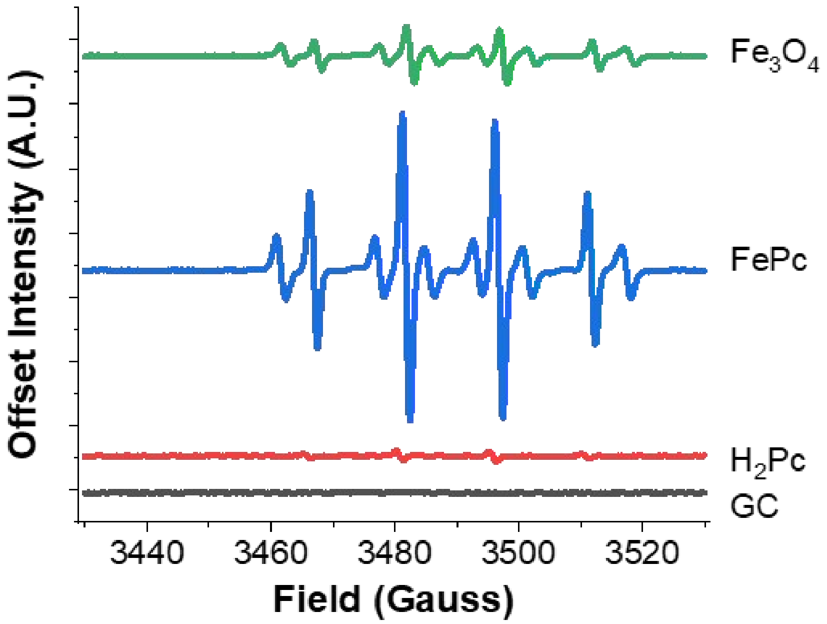

Figure S11 – ESR of model systems. ESR spectra recorded for each model system after two minutes of electrolysis at 0 V in an oxygen saturated acidic electrolyte containing 50 mM DMPO. The peak intensities were not calibrated using an internal standard, and so there could be variability in the quantity of ROS in solution from experiment to experiment. The SECM results should be consulted for comparative ROS generation quantities.

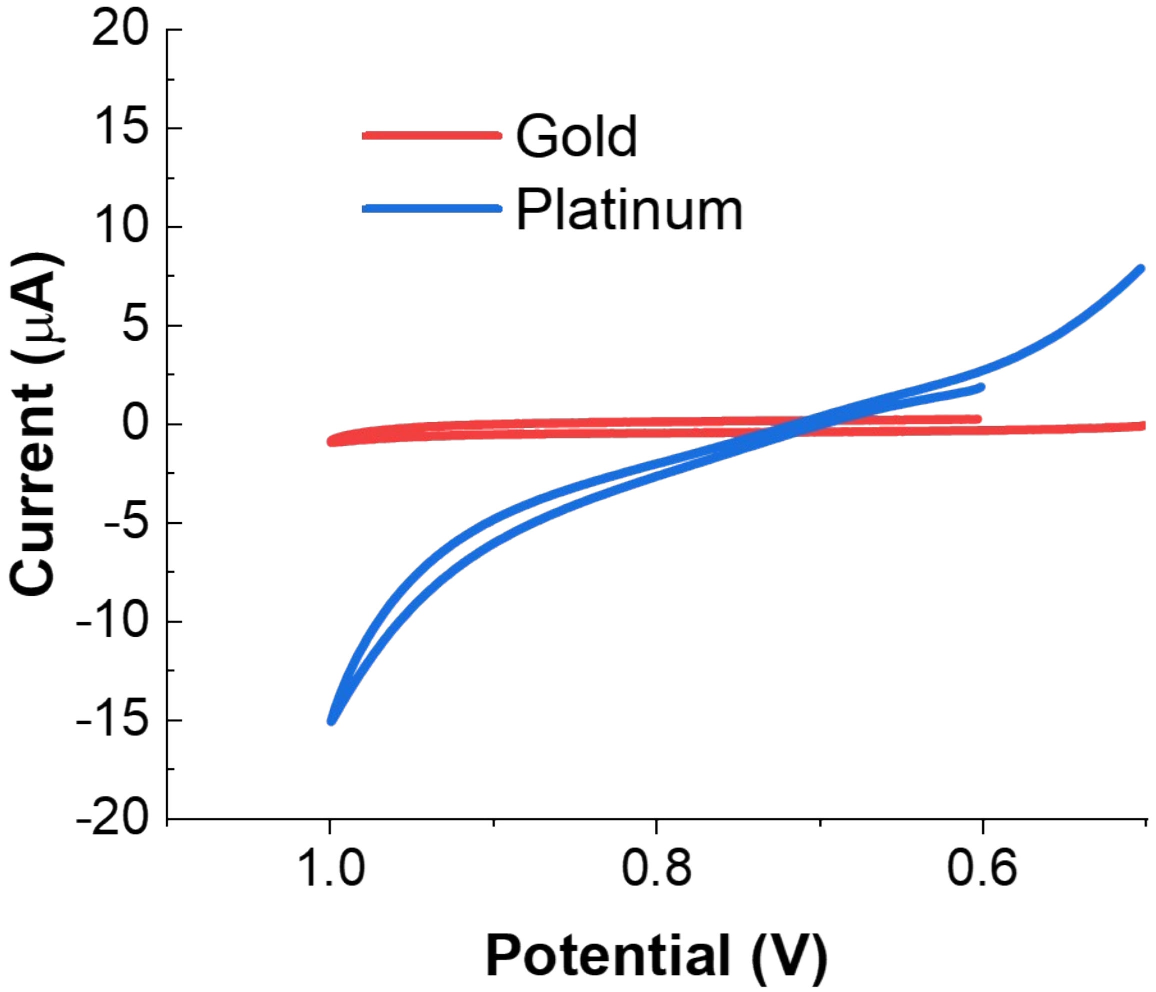

Figure S12 – H_2O_2 at gold and platinum electrodes. Cyclic voltammograms were recorded in a 0.5 M H_2SO_4 electrolyte solution open to air containing 1 mM of H_2O_2. Each voltammogram was measured using a 3 mm diameter working electrode of either polycrystalline gold or platinum. As has been shown in the literature, only the platinum electrode is capable of electrocatalytically oxidizing the H_2O_2, whereas the gold shows no response.[4] This is why gold was used as the tip material during the hydroxyl radical detection experiments.

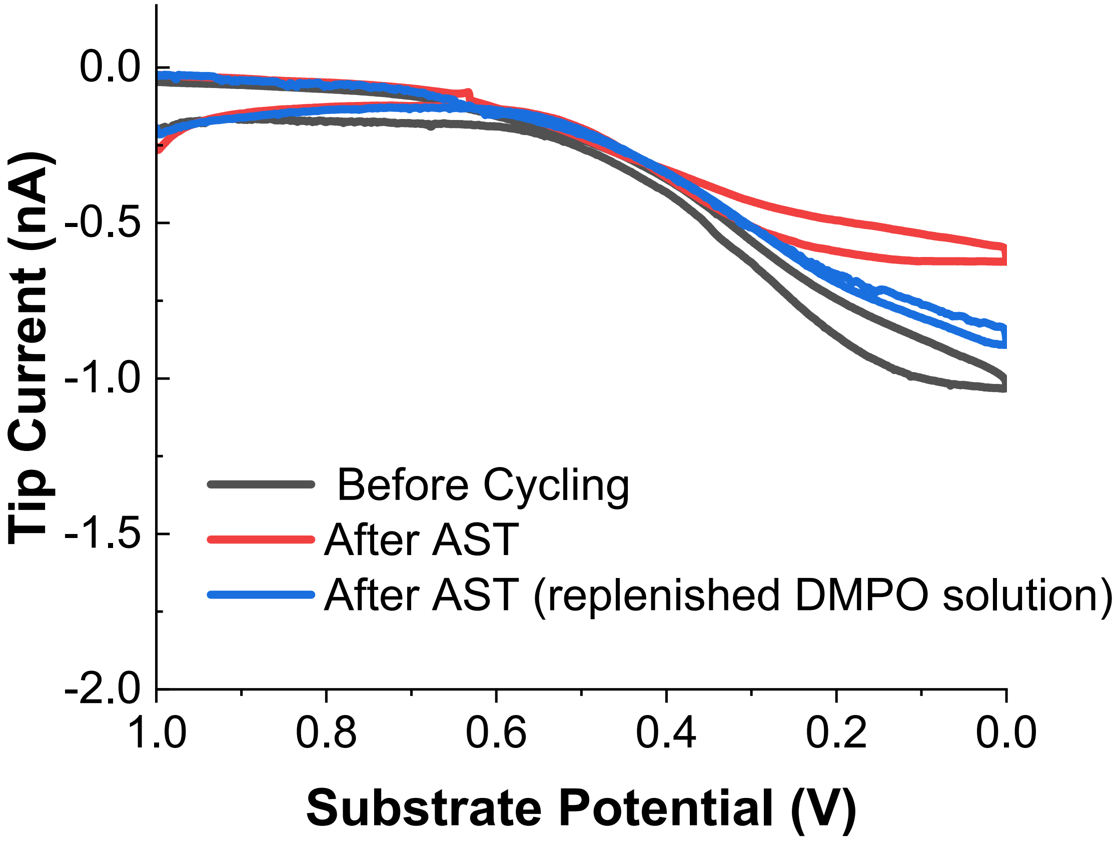

Figure S13 – DMPO solution regeneration after AST. After the accelerated stress test monitoring for OH radicals (**Figure 3A**), the DMPO solution by uncycled DMPO electrolyte. This was done, since during the long degradation cycles (as in **Figure S9A**), significant quantities of the DMPO initially present were either consumed (converted to DMPO-OH), or possibly degraded. The significant reduction of bulk DMPO concentration could affect the scavenging efficiency and lead to a lower tip current response than normally expected. This is confirmed by the partial recovery of tip current observed after changing solutions (blue trace). Comparing the replenished AST values with those obtained after H_2O_2 cycling shows that significantly less tip current was lost after degradation during the DMPO experiments. (**Figure 3B**)

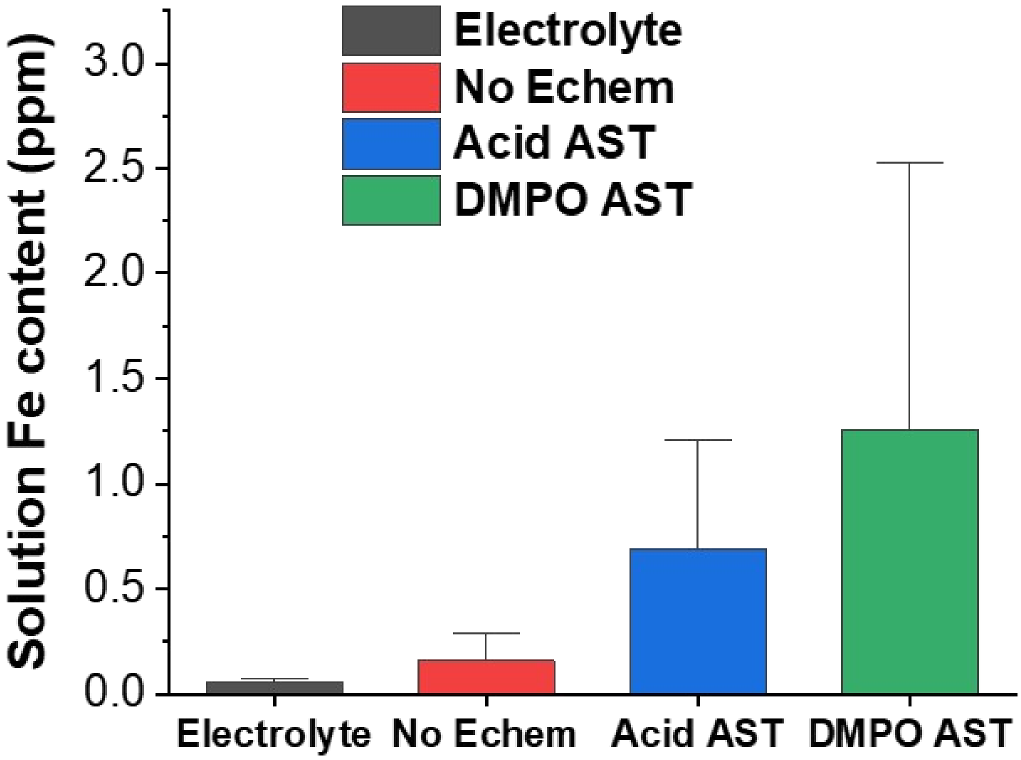

Figure S14 – Solution iron content (ICP-MS). To probe the amount of Fe dissolved from the catalyst under different conditions, ICP-MS was performed on samples taken under four different conditions: pure electrolyte, electrolyte exposed to the catalyst at OCP under oxygen saturation, electrolyte after an accelerated stress test (as in **Figure S9**), and electrolyte containing 50 mM DMPO after an accelerated stress test. Each experiment was performed in triplicate on different samples with different solutions.

References

1. A. Serov, M. J. Workman, K. Artyushkova, P. Atanassov, G. McCool, S. McKinney, H. Romero, B. Halevi and T. Stephenson, *J. Power Sources*, 2016, **327**, 557–564.
2. Q. Jia, N. Ramaswamy, U. Tylus, K. Strickland, J. Li, A. Serov, K. Artyushkova, P. Atanassov, J. Anibal, C. Gumeci, S. C. Barton, M.-T. Sougrati, F. Jaouen, B. Halevi and S. Mukerjee, *Nano Energy*, 2016, **29**, 65–82.
3. C. Lefrou and R. Cornut, *ChemPhysChem*, 2010, **11**, 547–556.
4. Y. Shen, M. Trauble, G. Wittstock, *Anal. Chem.* 2008, **80,** 3, 750-759.
